# New insertion method of transnasal ileus tube for small bowel obstruction: Anterior balloon method

**DOI:** 10.1371/journal.pone.0207099

**Published:** 2018-11-21

**Authors:** Daisuke Yamaguchi, Kei Ikeda, Yuki Takeuchi, Rikako Kinoshita, Toru Higuchi, Hiroko Fukuda, Naoyuki Tominaga, Tomohito Morisaki, Keisuke Ario, Seiji Tsunada, Hisako Yoshida, Kazuma Fujimoto

**Affiliations:** 1 Department of Gastroenterology, National Hospital Organization Ureshino Medical Center, Ureshino, Japan; 2 Department of Internal Medicine, Saga Medical School, Saga, Japan; 3 Department of Medical Statistics, Osaka City University Graduate School of Medicine, Osaka, Japan; University of Manitoba, CANADA

## Abstract

**Background:**

Small bowel obstruction (SBO) is usually caused by postoperative adhesions and malignant disease, and decompression is effective for SBO. Our previous case report suggested that a new transnasal ileus tube insertion method, the anterior balloon method (ABM), could achieve decompression for adhesive SBO.

**Aims:**

The study aimed to investigate the effectiveness of a new method for inserting transnasal ileus tubes in patients with SBO.

**Methods:**

Altogether, 134 patients with small bowel obstruction treated from January 2011 to December 2017 were reviewed. The patients were categorized into two groups: those with the new method that inserts an anterior balloon (ABM group: 52 patients, 2014–2017) versus those with the ordinary insertion method (OIM group: 82 patients, 2011–2014).

**Results:**

The patients’ characteristics and symptoms on admission were similar in the ABM and OIM groups. Adhesions were the main cause of ileus in the two groups. The insertion time duration was significantly shorter in the ABM group than in OIM group (28.4 ± 9.1 vs. 33.5 ± 13.0 min; p = 0.01). The ABM group also had significantly longer tubes than OIM group (222.4 ± 32.2 vs. 157.4 ± 31.7 cm; p < 0.001), which resulted in a significantly shorter time until clinical symptoms were relieved in ABM group. There were no significant differences in adverse events between the two groups.

**Conclusions:**

The ABM group had shorter insertion duration and longer tubes than those of OIM group. The ABM might become a preferred therapeutic choice to achieve decompression in patients with SBO.

## Introduction

Small bowel obstruction (SBO) is usually caused by postoperative adhesions, which develop in about 95% of adult patients after abdominal surgery [1]. Patients with this partially adhesive SBO are subjected to conservative management, which includes fasting, intravenous hydration, and decompression of the obstruction with a transnasal ileus tube [2]. Gastrointestinal decompression is an effective approach to treat patients with acute bowel obstruction without indications of strangulation [3]. As has been reported, decompression with an ileus tube has achieved favorable outcomes, including reduced edema, improved circulation of the involved intestine, and correction of intestinal kinking [4,5].

Decompression is effective for SBO caused by adhesive and/or malignant disease [6–8]. Decompression with an ileus tube is acceptable for all SBO patients who do not have a strangulation obstruction or other contraindications [9]. Difficult insertion of the ileus tube frequently results in a long procedure time, severe patient distress, and increased X-ray exposure of the patient and operator.

Our previous case report suggested that a new transnasal ileus tube insertion method, the anterior balloon method (ABM), could achieve decompression for adhesive SBO [10]. The aim of this retrospective evaluation was to compare the clinical outcomes of insertion with the ABM versus that with the ordinary insertion method (OIM) to achieve decompression for patients with SBO.

## Materials and methods

### Patients

A total of 134 consecutive patients with SBO who underwent ileus tube insertion at the National Hospital Organization Ureshino Medical Center from January 2011 through December 2017 were included in this retrospective evaluation. Patients >20 years of age who fulfilled the following criteria were candidates for the study: (1) presence of clinical symptoms and physical signs arising from acute bowel obstruction; (2) a diagnosis of SBO based on abdominal plain films and computed tomography (CT) scans and confirmed by at least two attending radiologists; (3) admission to the hospital within 24 h after the onset of the bowel obstruction. We excluded patients who (1) were suspected to have strangulated obstruction with symptoms of severe or sudden abdominal pain, had asymmetric abdominal distension or isolated swollen bowel loops, or were in shock; (2) required an immediate operation; or (3) had been treated in other hospital(s) before admission.

Informed consent for the procedures was obtained from all patients. The present study was conducted according to the Ethical Guidelines for Medical and Health Research Involving Human Subjects. Written informed consent for the procedures was obtained from all patients. The objectives, benefits, and harms of this study were explained verbally to the patients with the record in the hospital electronic medical chart. The present study was conducted according to the Ethical Guidelines for Medical and Health Research Involving Human Subjects. This study protocol and this consent procedure were approved by the Ethics Review Committee of the National Hospital Organization Ureshino Medical Center (approval number 17–31).

### Instruments

**Fig 1:**
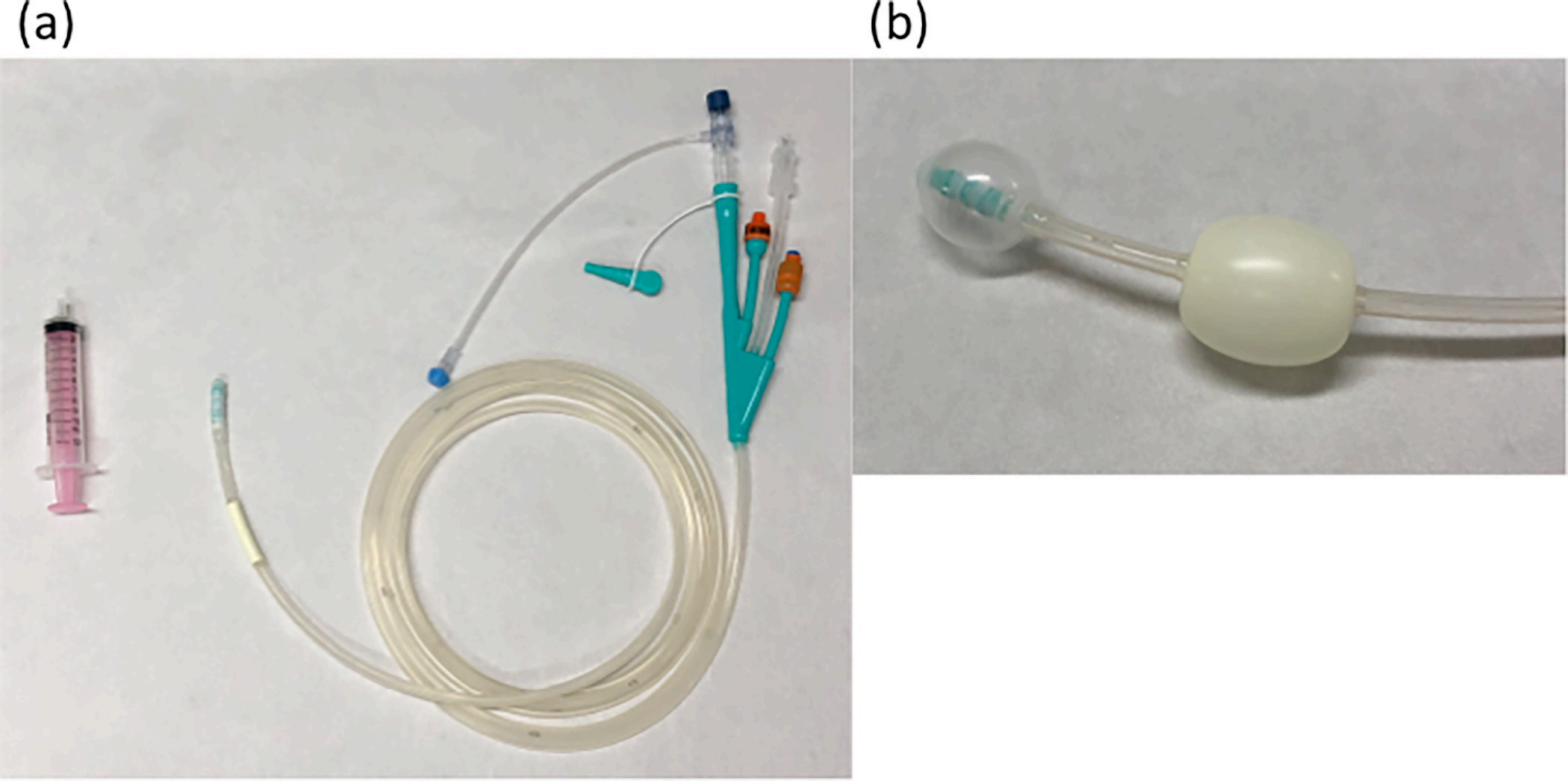
Instruments of ileus tube. (**a**) Appearance of the ileus tube (CLINY double-balloon type; Create Medic Co., Ltd, Tokyo, Japan) and the 10-ml syringe. (**b**) The tube had both anterior and posterior balloons at its tip.

The ileus tube (CLINY double-balloon type; Create Medic Co., Ltd., Tokyo, Japan) was 300 cm in length with an outer diameter of 16 Fr. It had both an anterior and a posterior balloon at the tip, a guidewire channel, and an injection channel with an anti-reflux valve (Fig 1) The hydrophilic guidewire was 1.32 mm in diameter and 450 cm long. A transnasal endoscope (GIF-XP260N; Olympus, Tokyo, Japan) was used in all patients during insertion of the ileus tube.

### Insertion of ileus tube

**Fig 2:**
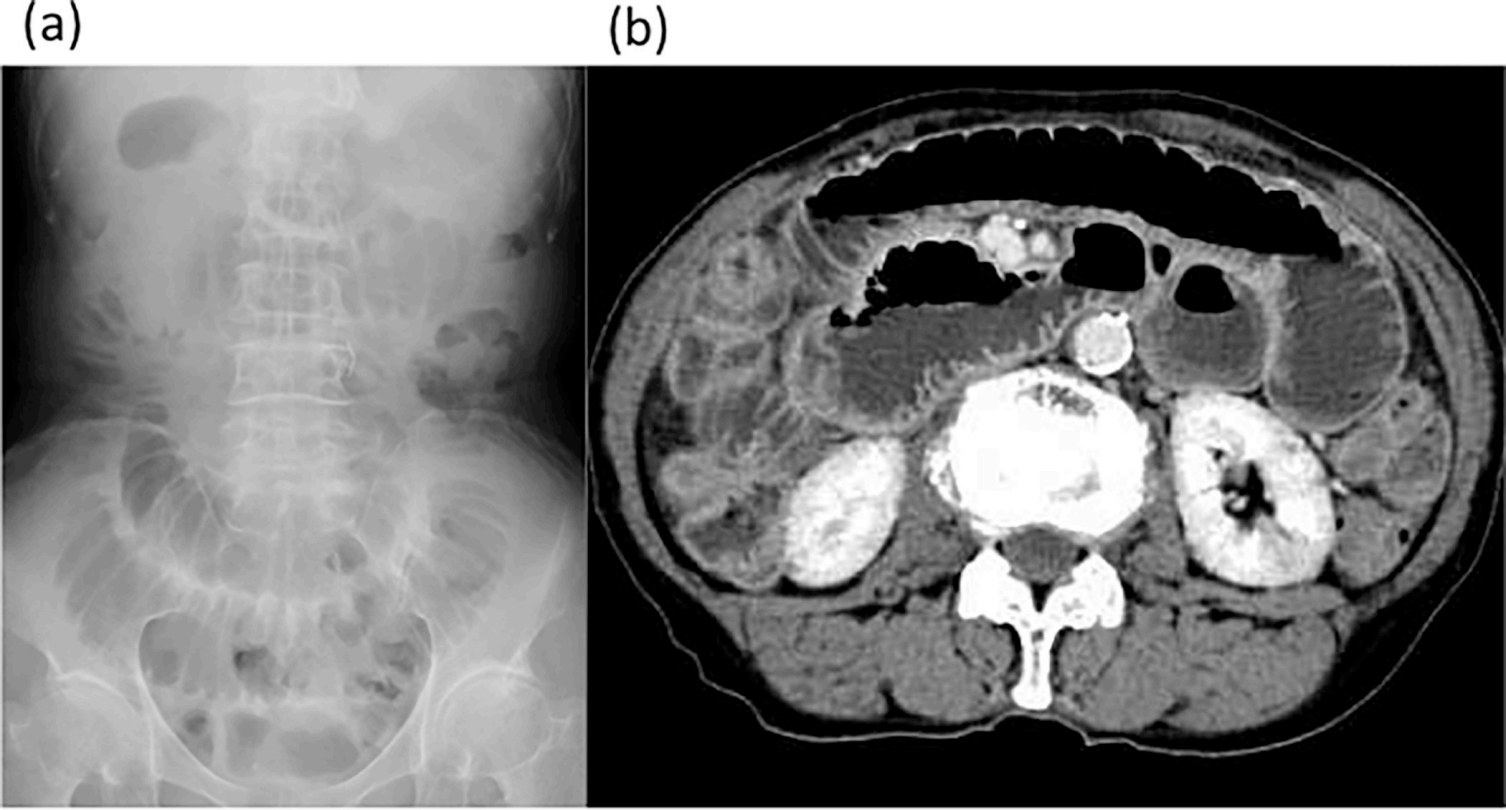
Radiography of acute bowel obstruction. Abdominal plain radiography (**a**) and abdominal computed tomography (**b**) of the adhesive small bowel obstruction.

On admission, each patient underwent abdominal plain film radiography and computed tomography (CT) to confirm the presence of acute bowel obstruction (Fig 2). To achieve decompression for adhesive SBO, the ileus tube was inserted using a transnasal endoscope. The endoscope was moved into the descending duodenum for insertion of the guidewire. After endoscope removal, the ileus tube was inserted into the duodenum through the guidewire.

The OIM was applied to patients from January 2011 through December 2014. An ileus tube guidewire was used to prevent the tube from twisting, and the tube was inserted into the jejunum by following a stiff guidewire under radiographic guidance. After the guidewire was pulled out, 15 ml of sterile water was injected into the posterior balloon, and bowel decompression was initiated. The tube was propelled by bowel peristalsis until decompression was completed. After insertion of the ileus tube, intermittent suction was performed to reduce intraluminal pressure in the small bowel. Abdominal plain films were obtained every other day to evaluate the progress of the tube and the degree of decompression. Before removing the ileus tube, water-soluble contrast medium was administered through the ileus tube to determine (1) the cause of the SBO, (2) whether the obstruction was partial or complete, and (3) whether it was completely relieved by the nonsurgical treatment. After removing the ileus tube, the patient was given a meal and discharged with no recurrence of the symptoms. No patient in the present study was re-admission with the recurrent symptoms within one month.

**Fig 3:**
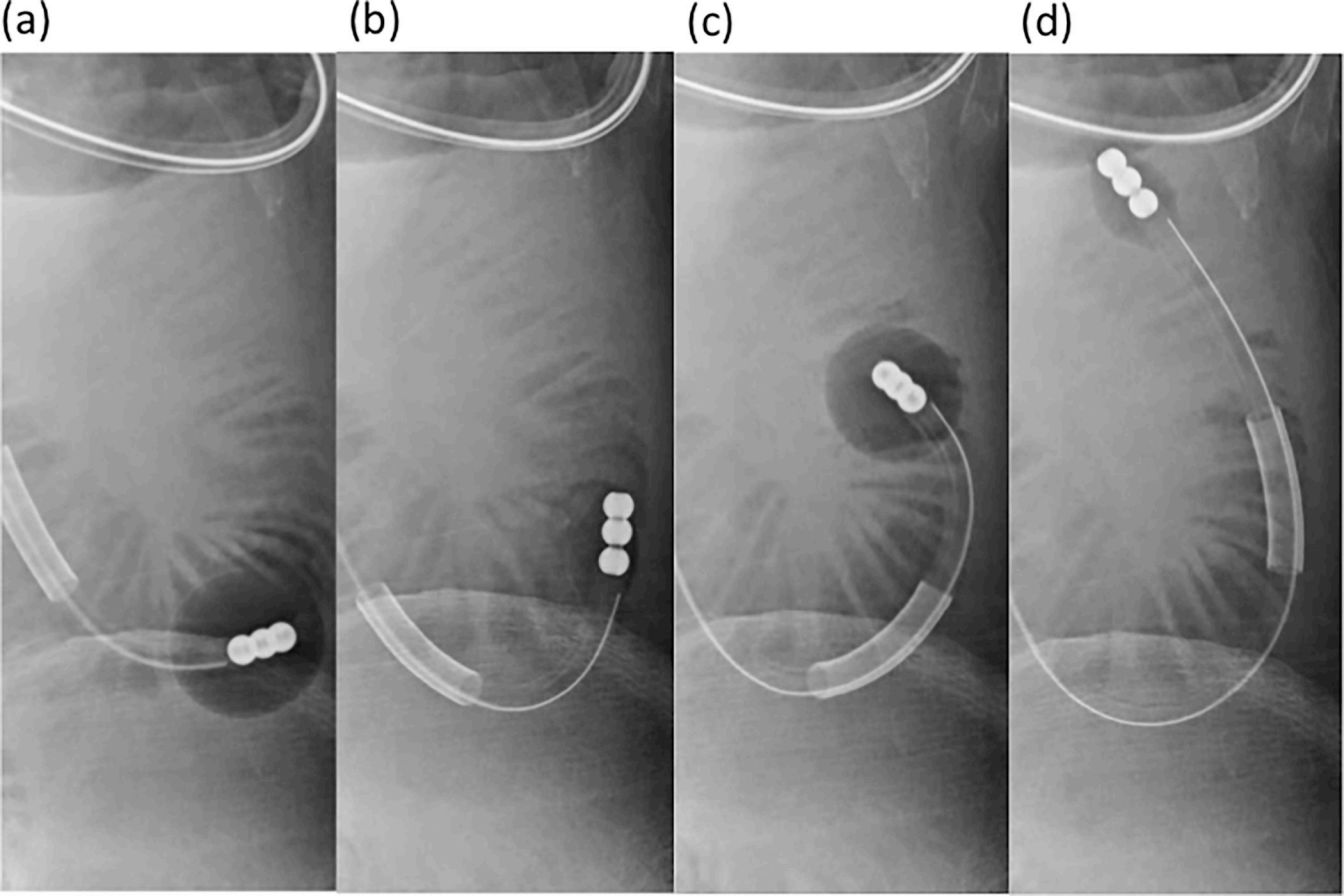
The process of ABM. Air was injected into the anterior balloon of the ileus tube (**a**) and then quickly suctioned out (**b**). (**c**, **d**) Ileus tube was advanced to the distal side of the small bowel by continuously repeating this method of air injection and removal.

As indicated in our previous case report [10], the new effective transnasal ileus tube insertion method—the ABM—was used from January 2014 through December 2017. The ABM ileus tube had anterior and posterior balloons. The trans-nasal endoscope was moved into the descending duodenum for insertion of the guidewire. After the endoscope removal, the ileus tube was inserted into the duodenum through the guidewire in the same way of the OIM. After inserting the tube into the duodenum, the anterior balloon was injected with 10 mL of air using a 10-ml syringe, which was then quickly suctioned. The process was continuously repeated until the tube reached close to the point of obstruction (Fig 3 and S1 Video). The intermittent suction was to reduce intraluminal pressure in the small bowel in a manner similar to that which occurred with the OIM.

The principle of the ABM is as follows: The anterior balloon is inflated to maintain a stable position, and the ileus tube is then bent by pushing. When the anterior balloon is deflated, the ileus tube advances to the distal aspect of the small bowel by the recovery force of the guidewire in the ileus tube, effectively straightening the bent ileus tube.

### Outcome measurement

The outcomes of the OIM and ABM groups were compared. During the observation period, nine endoscopists had inserted the ileus tubes. The distribution of the endoscopists was similar in the OIM and ABM groups. The following patient information was reviewed at the time of ileus tube insertion: age, sex, body mass index, performance status, previous abdominal operative history, cause of the ileus, and laboratory data. The causes of the ileus were classified as adhesive, paralytic, malignant, dietary, and other. The collected laboratory data included white blood cell counts and the hemoglobin, C-reactive protein, total protein, and albumin levels. Symptoms on admission—abdominal pain, abdominal distension, vomiting, defection—were recorded.

The patient’s information—i.e., duration of the insertion (‟insertion time”), length of the insertion tube, insertion by a trained endoscopist or a trainee—was reviewed during the ileus tube insertion. The insertion time was calculated as the time interval from the start of trans-nasal endoscopy to the finish of the ileus intubation. After ileus tube insertion, the following patient data were collected: insertion time, conservative treatment, surgery after the insertion tube, duration of surgery, resumption of meals, duration of hospitalization, and complications. The timing of the alleviation of symptoms was recorded (e.g., disappearance of abdominal pain, disappearance of distension, appearance of defecation). Using a visual analog scale (VAS) with VAS of 0 indicating no pain and 10 indicating on the full scale of the admission pain, the abdominal pain disappearance was defined as VAS of 0–1 assessed by the attending doctor. Distension disappearance was defined as clinical improvement of palpation and radiological disappearance of air-fluid levels.

Therapeutic effectiveness in the two groups was defined as clinical or radiologic improvement, relief of abdominal symptoms, decreased drainage volume, disappearance of air–fluid levels, or reduced gas and fluid in bowel loops. When both the clinical improvement of abdominal symptoms and radiologic improvement of air–fluid levels were confirmed, the decision was made to remove ileus tubes and to resume meals. If the patient presented no improvement 72 h after decompression or progressed into strangulation, surgery such as partial intestinal resection and anastomosis, enterolysis, or stoma creation was performed. After removing the ileus tube, the patient was given a meal and discharged with no recurrence of the symptoms for around 2 days.

### Statistical analysis

Results are expressed as means ± standard deviation. The χ^2^ test was used to identify differences in the effectiveness rate between the two groups. Student’s t test was used for unpaired data to determine differences in means between the two groups. A multivariate logistic regression was built to assess predictors of success cases with explanatory variables of age, sex, performance status, causes of ileus, Insertion time, length of insertion tube, insertion by trainees and insertion method. All statistical analyses were performed using JMP version 13.0.0 (SAS Institute, Tokyo, Japan).

## Results

Among the 134 patients in whom an ileus tube was inserted, 52 were in the ABM group and 82 in the OIM group. Patients’ characteristics of the two groups are shown in Table 1. There were no significant differences between the groups regarding the clinical characteristics or the laboratory variables documented on admission. The main types of ileus represented in the two groups were adhesive ileus (73.9%), paralytic ileus (15.7%), and malignant ileus (6.0%). The patients’ symptoms on admission—fever, abdominal pain, abdominal distension, vomiting, defecation—were similar in the two groups.

**Table 1:** Characteristics of the patients with ileus tube insertion.

Characteristics	ABM group	OIM group	p
Number of patients	52	82	
Age (years)	72.0 ±12.3	72.9 ±13.5	0.69
Male sex	32 (61.5%)	48 (58.5%)	0.70
Body mass index (kg/m^2^)	19.6 ±3.3	20.3 ±3.0	0.20
Performance status (0–2)	43 (82.7%)	66 (80.5%)	0.47
History of abdominal surgery	36 (72.0%)	61 (74.4%)	0.83
Causes of ileus			
Adhesive	39 (75.0%)	60 (73.2%)	0.10
Paralytic	9 (17.3%)	12 (14.6%)	
Malignant	2 (3.9%)	6 (7.3%)	
Dietary	2 (3.9%)	2 (2.4%)	
Other	0	2 (2.4%)	
Symptoms at admission			
Fever	2 (3.9%)	2 (2.4%)	0.64
Abdominal pain	42 (80.8%)	70 (85.4%)	0.49
Abdominal distension	39 (75.0%)	61 (74.4%)	1.00
Vomiting	37 (71.2%)	55 (67.1%)	0.70
Defecation	1 (1.9%)	5 (6.1%)	0.40
Laboratory data			
White blood cell count (/μl)	9407.7 ± 3397.4	9480.0 ±3754.5	0.91
Hemoglobin (g/dl)	12.7 ± 2.3	12.9 ±2.1	0.68
C-reactive protein (mg/dl)	3.2 ± 5.2	2.1 ±3.8	0.18
Total protein (g/dl)	6.9 ± 0.9	6.8 ±0.9	0.60
Albumin (g/dl)	3.7 ± 0.8	3.8 ±0.7	0.72

Results are given as the mean ± SD or the number (%) unless otherwise stated.

Table 2 shows the treatment outcomes of ileus tube insertion in the two groups. The ABM group had a significantly shorter insertion time and significantly longer insertion tubes than the OIM group. The ABM group experienced significantly shorter time until relief of clinical symptoms including the disappearance of abdominal pain, disappearance of distension, and appearance of defecation.

**Table 2:** Treatment outcomes of ileus tube insertion.

Parameters	ABM group	OIM group	p
Number of patients	52	82	
Insertion time (min)	28.4 ± 9.1	33.5 ± 13.0	0.01
Insertion tube length (cm)	222.4 ± 32.2	157.4 ± 31.7	<0.001
Use of transnasal endoscopy	52 (100%)	82 (100%)	
Insertion by trainees	38 (73.1%)	48 (58.5%)	0.10
Duration of tube insertion (days)	4.2 ±3.5	5.9 ± 5.3	0.03
Conservative treatment	43 (82.7%)	64 (78.0%)	0.66
Surgery after tube insertion	9 (17.3%)	18 (22.0%)	0.66
Duration of surgery (days)	1.2 ±4.2	1.7 ± 4.2	0.54
Outcomes of symptoms			
Abdominal pain disappearance	2.0 ±1.6	3.1 ± 4.1	0.03
Distension disappearance	2.0 ±1.5	3.1 ± 4.1	0.03
Defecation appearance	2.7 ±1.6	5.2 ± 4.6	<0.001
Resumption of meals (days)	4.4 ±3.5	6.0 ± 5.2	0.03
Hospitalization (days)	21.7 ±28.4	23.4 ± 27.5	0.74
Adverse events			
Reinsertion	0	0	
Bleeding	0	0	
Perforation	0	0	
Mortality	0	0	

Results are given as the mean ± SD or the number (%) unless otherwise stated.

**Fig 4:**
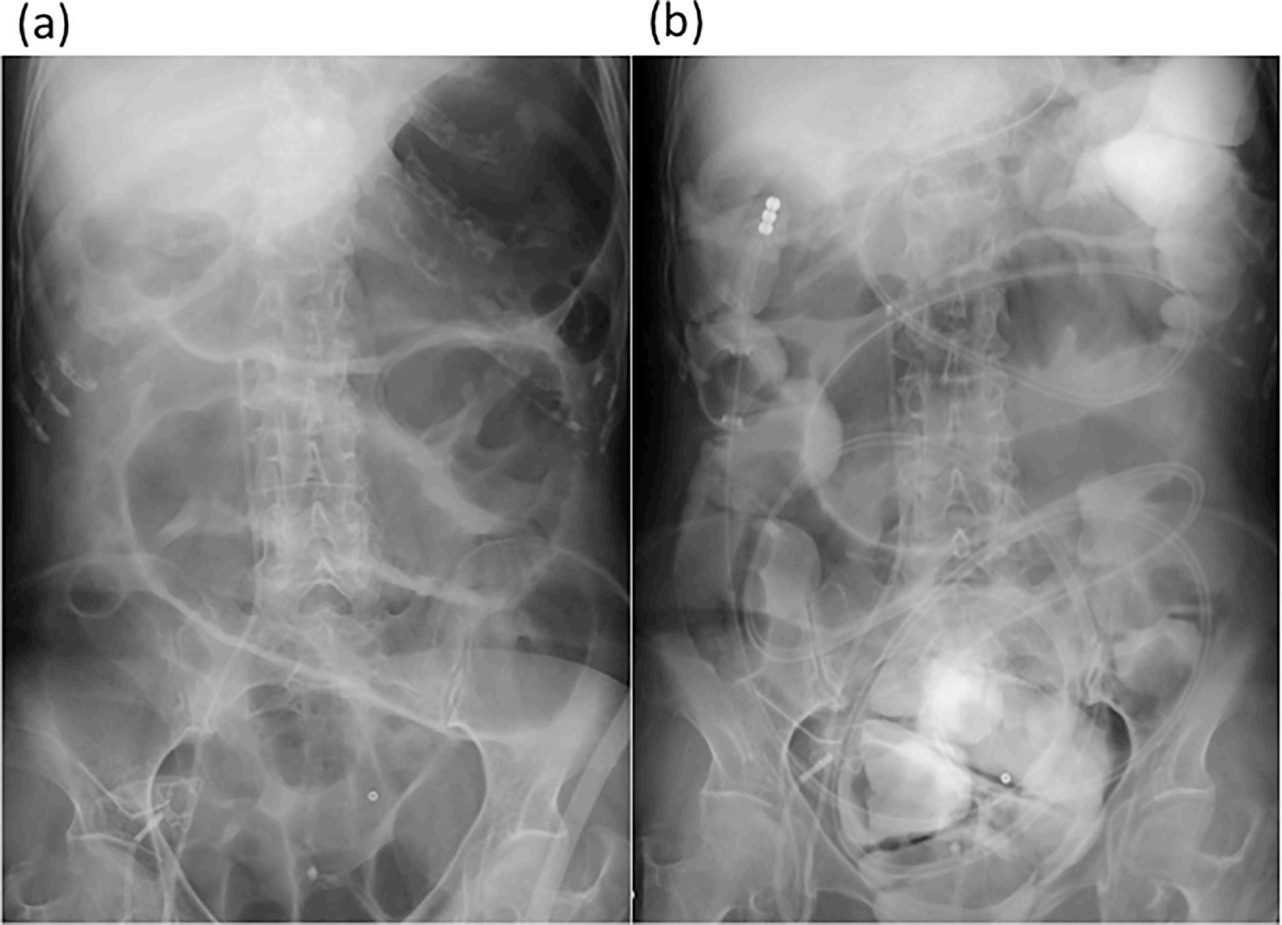
Radiological improvement after insertion of ileus tube. Abdominal radiograph on admission in a patient with adhesive small bowel obstruction (**a**) compared with the radiograph obtained 1 day after ileus tube decompression (**b**). The previous gas-filled or fluid-filled small bowel loops showed no evidence of distension, the air–fluid levels disappeared, and the tip of the ileus tube reached the colon.

After ileus tube decompression, 107 patients (81.1%) experienced clinical or radiographic relief (Fig 4), and the ileus tube could be removed (conservative treatment). Another 8 patients (6.0%) with malignant obstruction underwent surgery, and 19 patients (14.2%) with defined treatment failures underwent surgery to treat the obstruction. The surgery rate after tube insertion was similar in the two groups (17.3% vs. 22.0%). The conservative management and the surgical intervention were not different between the two groups.

The mean overall insertion time was shorter in the ABM group than in the OIM group (4.2 ± 3.5 vs. 5.9 ± 5.3 days; p = 0.03). After removing the ileus tube, meal resumption started significantly sooner in the ABM group (4.4 ± 3.5 vs. 6.0 ± 5.2 days; p = 0.03). There were no significant differences in adverse events due to ileus tube insertion between the two groups.

The result of the multivariate logistic regression analysis was indicated in Table 3. These explanatory variables had no influence on successes of conservative treatment of insertion ileus tube.

**Table 3:** Multivariate analysis of predictors of success in ileus tube insertion.

	Multivariate analysis			
Variables	Odds ratio	95% CI		p
Age, >70 y	2.07	0.76	5.67	0.14
Sex, male	0.88	0.34	2.27	0.79
Performance status, >2	0.79	0.25	2.53	0.69
Causes of ileus, Adhesive	0.67	0.22	2.00	0.46
Insertion time, >30 min	1.19	0.48	2.98	0.70
Length of insertion tube, >200 cm	0.81	0.24	2.75	0.73
Insertion by trainees	0.41	0.15	1.18	0.08
Insertion method, ABM	0.81	0.24	2.76	0.74

## Discussion

Decompression via ileus tube is a major noninvasive therapeutic approach in patients with clinical and/or radiographic SBO induced by several causes [11]. Since the 1930s, various tubes have been devised and used for gastrointestinal decompression [12–14], which has resulted in markedly reduced mortality associated with bowel obstruction [4,15]. The present study indicated that the success rate of conservative treatment with the ileus tube without serious adverse events was 81.1%, which is similar to previous reports [1,4,5]. The present study indicated three advantages of the new transnasal ileus tube insertion method (ABM) compared with OIM: (1) the increased length of the insertion tube; (2) the shorter insertion time; (3) quick relief of the clinical symptoms of SBO.

The ABM group had significantly longer insertion tubes than those used in the OIM group. The insertion method of the tube with fluoroscopic guidance was replaced with direct placement under endoscopic guidance, leading to greater safety and high success rates [5,16,17]. The present study with endoscopic insertion used a longer ileus tube in the ABM group. As indicated in previous studies, decompression with a long ileus tube to aspirate the intestinal contents decreased edema of the bowel wall [18], enhanced bowel motility, and prevented bacterial translocation [18,19]. Because the longer insertion tube in ABM group was automatically passed into the deeper portion of the intestine by the balloon transport, the tube came closer to the obstruction and might reduce the intraluminal pressure more effectively. Also, the longer tube in the ABM group facilitated radiographic diagnosis of bowel obstruction, which was helpful for identifying the point(s) of obstruction.

The insertion time of the ileus tube was shorter in the ABM group than in the OIM group. The insertion time in the ABM group was 28.4 ± 9.1 min, which was almost equivalent to the previous studies [20,21]. The insertion of a long tube was traditionally performed under the guidance of the X-ray, which induced many drawbacks including prolonged procedural time, severe patient distress, and increased X-ray exposure. Direct observation using the endoscopy made it much easier and quicker to guide the tube through the pyloric ring [22]. As indicated in the present study, ABM under the endoscopic guidance avoided the X-ray exposure resulting in improved safety for the medical staff and the patient without serious complications.

Clinical symptoms were relieved within a shorter time in the ABM group than in the OIM group, leading to a significantly shorter interval until meal resumption. In the present study, 27 patients (20.1%) underwent operation to treat the obstruction after insertion of the ileus tube. Cause of ileus was not a predictor of success of ileus tube insertion on the multivariate analysis and had no influence on surgical treatment for SBO. The surgeon inclined to choose conservative treatment for adhesive bowel obstruction [23]. As previously demonstrated, the patient who failed to respond 3 day after decompression, or had the ischemic bowel, or the drainage volume more than 500 mL on the third day was recommended to the surgical treatment [24]. The fairly prompt response within 48 h after decompression of the ileus tube avoided the surgical intervention [25,26], which supported advantage of ABM which performed effective decompression with the short duration.

Economically, the cost of this ileus tube was 85,000 yen which was a little bit expensive compared to the other ileus tubes (about 48,000–52,000 yen). The procedure and the instruments of ABM used in the present study are we available in most hospitals in Japan, which might be an advantage of ABM.

The present study did have limitations. It was a retrospective study, not a prospective controlled trial, and the number of the patients was limited to a single institution. Moreover, the present study did not remove the issue of the selection bias in the two tested groups. Thus, our results warrant confirmation.

In conclusion, ABM, a new ileus tube insertion method, is a therapeutic approach to relieving an SBO using easily available instruments and devices compared to OIM.

## Supporting information

S1 Video The video of ABM. The video indicates that the ileus tube advances to the distal side of the small bowel by continuously repeating the air injection/removal method.
(MP4)

## Abbreviations

ABM: anterior balloon method
OIM: ordinary insertion method
SBO: small bowel obstruction
CT: computed tomography
